# 

**Published:** 1908-01-18

**Authors:** 


CONTENTS.

| | PAGE |
|---|---|
| **Proprietary Preparations** ... ... ... ... ... ... | 407 |
| **Rat Extermination** ... ... ... ... ... ... ... ... | 408 |
| **Annotations—** | |
| Malaria in the Roman Campagna—Regulation of Fees in the Indian Medical Service—The "Albion Magazine" ... ... | 409 |
| **Medical Opinion and Movement** ... ... ... ... | 410 |
| **Hospital Clinics—** | |
| Syphilitic Disease of the Joints. By D'Arcy Power, F.R.C.S., Eng.... ... ... ... | 411 |
| A Note on Co-Existent Infectious Diseases. By J. T. C. Nash, M.D., etc. ... ... | 416 |
| **Points in Treatment—** | |
| Antistreptococcic Serum in Phthisis with Cavitation ... ... | 417 |
| Oxygen Inhalations should be Warmed ... ... ... ... | 418 |
| **Illustrative Cases—** | |
| A Case of Spontaneous Rupture of a Coronary Artery ... ... | 418 |
| **Points in Surgery—** | |
| Some Surgical Complications of Typhoid Fever ... ... ... | 419 |
| Some Diseases of the Male Genital System... ... ... ... | 420 |
| **Gynæcology—** | |
| Puerperal Fever in London in 1906 ... ... ... ... ... | 421 |
| **Laryngology and Rhinology—** | |
| Cancer of the Larynx ... ... ... ... ... ... ... | 422 |
| **Public Health and Hygiene—** | |
| Report of the Medical Officer of the Education Committee of the London County Council ... ... ... ... ... | 423 |
| Diagram of the Weekly Death Rate in 1907 ... ... ... ... | 424 |
| **The Practitioner's Relaxations and Hobbies—** | |
| Architecture and History ... ... ... ... ... ... | 425 |
| **Practical Notes on Diagnosis and Treatment** ... | 426 |
| **"The Hospital" Medical Book Supplement.—No. 3** ... ... ... ... ... ... ... | 427 |
| **Hospital Administration—** | |
| Current Hospital Topics ... ... ... ... ... ... ... | 431 |
| Isolation Hospital Expenses ... ... ... ... ... ... | 432 |
| News and Coming Events ... ... ... ... ... ... | 433 |
| The Graduates' Medical Schools ... ... ... ... ... | 433 |
| **Editor's Letter-Box—** | |
| One More Central Authority for Charity ... ... ... ... | 434 |

